# Duodenal obstruction - an unusual presentation of *Strongyloides stercoralis *enteritis: a case report

**DOI:** 10.1186/1749-7922-5-23

**Published:** 2010-08-10

**Authors:** Ruy J Cruz, Rodrigo Vincenzi, Bernardo M Ketzer

**Affiliations:** 1 Department of Surgery, University of Pittsburgh Medical Center, Pittsburgh, PA, USA; 2 Department of Surgery, University of Santo Amaro Medical School, Sao Paulo, Brazil

## Abstract

**Background:**

Intestinal obstruction is a poorly recognized and probably underreported complication of strongyloidiasis. We present herein an unusual case, of complete duodenal obstruction caused by *S. stercoralis*.

**Methods:**

A systematic review of the literature examining the clinical course, diagnostic methods, and outcome of this rare complication of strongyloidiasis was performed.

**Results:**

A 42-year-old woman presented with a 5-month history of abdominal pain, vomit, and weight loss. An abdominal CT scan showed an obstruction of the third part of the duodenum. Segmental intestinal resection was carried out and histopathology examination revealed heavy *Strongyloides stercoralis *infestation. Duodenal obstruction is a rare complication of *S. stercoralis *infection, with only 8 cases described in the literature since 1970. Most of the patients are males, middle-aged, and the diagnosis was made by duodenal aspirate/biopsy, or analysis of surgical specimen.

**Conclusions:**

Duodenal obstruction is an unusual, but potential fatal, complication of *S. stercoralis *infection. The large spectrum of clinical manifestation and lack of classic clinical syndrome make the final diagnosis of strongyloidiasis extremely difficult. A high index of suspicion, mainly in patients from endemic areas, is needed for correct and early diagnosis of this uncommon presentation of *Strogyloides stercoralis *enteritis.

## Background

Strongyloidiasis is a parasitic disease, caused by a nematode helminth, *Strongyloides stercoralis*. The true prevalence of *S. stercoralis *is likely underestimated because infection is often subclinical [1-3]. Currently, an estimated 100 million people are infected worldwide in more than 70 countries. Strongyloidiasis is endemic in Southeast Asia, Latin America, Sub-Saharan Africa, and parts of the southeastern United States [3-6]. Typically, the infection is asymptomatic or manifest as vague and unspecific gastrointestinal symptoms. However, disseminated infestation of infective larvae is associated with high mortality rates in immunocompromised patients [3,7].

Intestinal obstruction is a poorly recognized and probably underreported complication of strongyloidiasis. Herein, we report an unusual case, of complete duodenal obstruction caused by *S. stercoralis*. Additionally, we performed a systematic review of the literature examining the clinical course, diagnostic methods, management and outcome of this rare, but potential fatal complication of *S. stercoralis *infection.

## Methods

A review of literature was performed using the MEDLINE database in order to identify articles of duodenal obstruction caused by *Strongyloides stercolaris*. Inclusion was limited to cases reported in adults, and published in the English language since 1970. All the articles were systematically reviewed and only cases of confirmed duodenal obstruction were included in this report.

## Case presentation

A 42-year-old woman presented with a 5-month history of recurrent abdominal pain, nausea, post-prandial vomiting, intermittent diarrhea, and a 20 Kg (44 lb) weight loss. Her past medical history was unremarkable, except for an admission for pneumonia in the past year. On physical examination the patient was in poor clinical condition, malnourished, afebrile, with a blood pressure of 100/40 mmHg, pulse of 100 beats per minute and a respiratory rate of 24 breaths per minute. No lymphadenophaty was found. The lungs were clear and the heart was normal on auscultation. Abdominal examination revealed epigastric distention, without guarding or rebound tenderness. The spleen and liver were not palpated and a mild pedal edema was observed.

Stools tested for occult blood were positive, and negative for ova and parasites. Laboratory evaluation revealed a hematocrit of 39%, white blood cell count of 14.9 × 10^3^/L (bands 8%, neutrophils 73%, lynphocytes 12%, and eosinophils 0%), and platelet count of 600 × 10^3^/μL. Total serum protein and albumin levels were 2.9 g/dL and 1.2 g/dL, respectively. Serum creatinine was 2.5 mg/dL, BUN 118 mg/dL, and potassium 2.8 mMol/L. Liver function tests, amylase and lipase were within normal limits. She had a positive serology for toxoplasmosis (IgM antibody), but negative for HIV, and HTLV-1.

**Figure 1:**
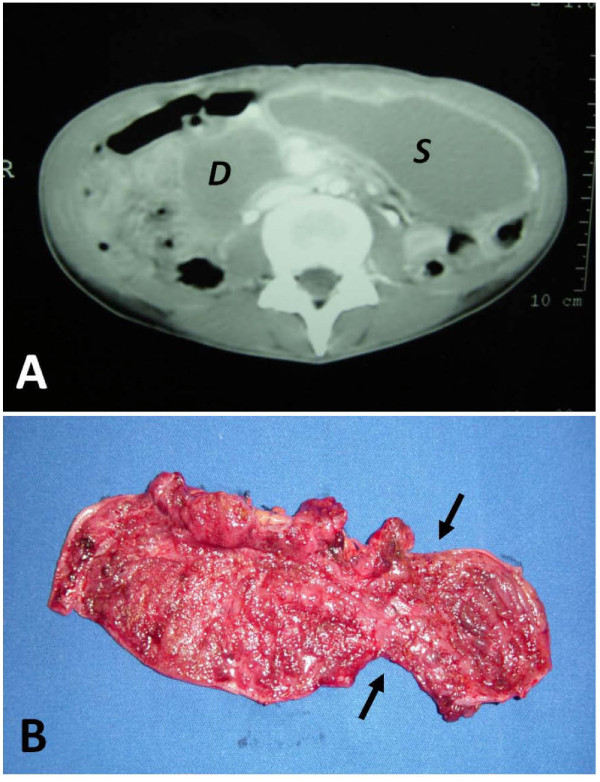
**(A) Abdominal CT scan showing a large dilation of stomach (*S*) and duodenum (*D*)**. (**B**) Severe inflammation, mucosal hemorrhage and focal ulcerations of duodenum and proximal jejunum. Black arrows show the point of obstruction.

A central line was established and fluid replacement was started. Broad-spectrum antibiotics were initiated for a possible intraabdominal infection/sepsis. An abdominal computed tomographic scan showed a marked gastric and duodenal distension, with a possible point of obstruction of the third part of the duodenum (Figure 1A). A nasogastric tube was placed for gastric decompression. Upper endoscopy was nondiagnostic due to a marked retention of alimentary residue in the stomach.

At this point we decided to start the patient on total parenteral nutrition and repeat the upper endoscopy in 48 hours. Despite clinical support, 24 hours after admission, the patient presented a significant worsening of the abdominal pain, fever, increasing white blood cell count, and intermittent hypotension requiring additional intravenous fluid bolus. Based on the abdominal CT findings, we suspected of the presence of a complicated submucosal duodenal tumor, such as a primary intestinal lymphoma or gastrointestinal stromal tumor, and decided to take the patient to the operating room.

**Figure 2:**
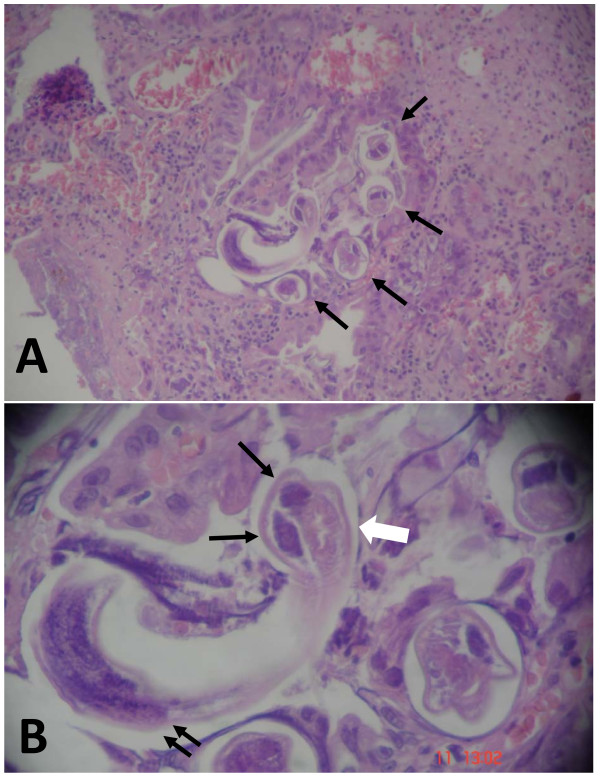
**Histopathological examination of the duodenal mucosa (hematoxylin-eosin staining)**. **(A) **Cross-sections of *Strongyloides *larvae within the intestinal mucosa (arrows) associated with diffuse eosinophil and plasma cell infiltration. **(B) **Higher magnification showing a female *Strongyloides stercolaris *ovaries (arrows) and intestine (white arrow). A longitudinal section of *S. stercolaris *larva can also be observed (double arrow).

She underwent an exploratory laparotomy that showed diffuse thickening and edema of the proximal small bowel, and a severe stenosis of the third part of the duodenum. Resection of the narrowed segment was carried out and an end-to-end duodenojejunostomy was performed. The resected specimen showed a severe inflammatory process, associated with mucosal ulceration and hemorrhage (Figure 1B). Histopathology examination revealed severe inflammation of the intestinal wall with heavy infestation of *Strongyloides stercoralis *(Figures 2A, and 2B). The patient was sent to the intensive care, antibiotics were continued, and treatment for disseminated strongyloidiasis with a combination therapy of ivermectin at a dose of 200 mcg/kg daily and albendazole 400 mg twice a day was started. Despite adequate clinical support, the patient died of septic shock seven days after exploratory laparotomy.

## Discussion

Strongyloidiasis is a common intestinal infection caused by two species of the nematode *Strongyloides*. The most common and clinically important pathogenic species in humans is *Strongyloides stercoralis*. The other specie, *Strongyloides fuelleborni*, is found sporadically in Africa and may produce limited infections in humans [3,8]. Strongyloidiasis was first described in 1876, in French colonial troops suffering from diarrhea in Vietnam [9].

The complete elucidation of the parasite's life cycle occurred 50 years after its identification. This fact is probably related to the unique and complex life cycle of the parasite. *Strongyloides stercoralis *larvae exist in two forms: free-living rhabditiform and filariform infective larvae. The cycle starts with the infectious filariform larvae penetrating the skin and traveling via lymphatics or bloodstream to the lungs. After penetrating in the alveoli the larvae continue to migrate up to the airways until they are swallowed. In the duodenum and proximal jejunum the larvae mature into adult females which live threaded in the intestinal mucosa. The larvae can produce up to 40 eggs a day by mitotic parthenogenesis (*i.e*., asexual reproduction where development of embryos occurs without fertilization by a male). Once these eggs hatch, rhabditiform larvae are released. These larvae can either passed in the stools, continuing the soil based cycle, or can cause autoinfection. The autoinfection occurs when the rhabditiform larvae prematurely become the infective filariform larvae in the intestinal lumen, and penetrate in the intestinal mucosa or perianal skin (internal and external autoinfection, respectively). In either case the infective larvae migrate to the lungs and restart the cycle previously described [1,3,7]. The autoinfection phenomenon allows *S. stercoralis *to persist and replicate within a host for decades, with the longest reported period being 65 years [10].

The term "disseminated disease" is used to define when the infective larvae migrate, from the intestine, in massive numbers not only to the lungs but to other organs not involved in the normal helminthic life cycle. In disseminated strongyloidiasis, the mortality rate can be as high as 70-90% [3]. Several risk factors are associated with the development of disseminated strongyloidiasis, including (1) immune deficiency, (2) hematologic malignacy, (3) steroids administration, (4) HTLV-1 infection, (5) chronic alcoholism, (6) renal failure, (7) transplantation, among others [11]. In disseminated disease, translocation of enteric bacteria may occur, leading to Gram-negative sepsis and/or meningitis. The enteric microorganism can either enter the circulation through intestinal ulcers or be carried by the infective filariform larvae.

Approximately, half of *Strongyloides *infections are asymptomatic [1,3]. Clinical presentation is extremely variable reflecting the complex life cycle of the parasite. When symptoms develop, gastrointestinal complaints are common. Symptoms are vague and nonspecific and include anorexia, nausea, vomiting, weight loss, abdominal pain, flatulence, and diarrhea. Less frequently, malabsorption syndromes, paralytic ileus, intestinal obstruction and gastrointestinal bleeding, may occur [1-3]. Pulmonary symptoms are rare in uncomplicated strongyloidiasis, but cough and wheezing may be part of initial presentation (Löffler's syndrome). In disseminated disease respiratory symptoms become more prominent and include dyspnea, tachypnea, pleuritic pain, pleural effusion, and hemoptysis [1,2,6]. Larva currens (racing larvae) is the pathognomonic cutaneus manifestation of *Strongyloidis *infection that usually occurs during an external autoinfection episode. The serpiginous urticarial rash is caused by rapid (approximatelly 15 cm/h) moving of *Strongyloides stercoralis *larvae from the anal area down the upper thighs [3,12].

Duodenal obstruction is an extremely rare complication of strongyloidiasis, with eight cases reported in the medical literature. Table 1 summarizes all the reported cases of duodenal obstruction caused by *Strongyloides stercolaris *since 1970 [9,13-18]. Two mechanisms have been implicated in the duodenal obstruction due to *S. stercoralis*. First, the obstruction would be related to a severe mucosal edema and inflammation with significant narrowing of duodenal lumen. Second, an extrinsic compression of the duodenum by the superior mesenteric neurovascular bundle could be responsible for the obstructive symptoms. Several mechanisms are proposed to explicate the extrinsic duodenal compression (*i.e*. superior mesenteric artery/Wilkie's Syndrome) in patients with strongyloidiasis, including severe weight loss, duodenal distention, mesenteric lymphatic dilation, and increase in the diameter of superior mesenteric vessels [15,16,19].

**Table 1:** Literature review of duodenal obstruction caused by *Strongyloides stercoralis *infection (1970-2010).

Author	Year	Age	Gender	Country	Associated disease	WBC/eosinophils	Surgery	Diagnosis	Treatment	Outcome
Cohen & Spry^13^	1979	40	M	England	lymphoma	16.500/4%	SB resection	DA, EGD+bx	thiabendazole *	Dead
Zyngier et al.^14^	1983	30	M	Brazil	no	NR/0%	gastrojejunostomy	GA, sputum	thiabendazole †	Alive
Lee & Terry^15^	1989	15	M	Jamaica	no	4.400/NR	no	stool analysis	thiabendazole ‡	Alive
	1989	19	F	Jamaica	no	10.000/NR	no	DA	thiabendazole	Alive
Friedenberg et al.^16^	1999	40	M	USA	HTLV-1 infection	35.500/1%	no	EGD+bx	thiabendazole	Dead
Harish et al.^9^	2005	45	M	India	no	12.000/14%	no	DA, EGD+bx	ivermectin	Alive
Suvarna et al.^17^	2005	70	M	India	no	11.000/(220/μL)	no	EGD+bx	ivermectin #	Alive
Juchems et al.^18^	2008	63	M	Germany	no	10.500/NR	partial gastrectomy	surgical specimen	ivermectin	Alive
Current case	2010	42	F	Brazil	no	14.900/0%	duodenal resection	surgical specimen	ivermectin + albendazole	Dead

NR, not reported; WBC, white blood cell count; DA, duodenal aspirate; GA, gastric aspirate; EGD, esophagogastroduodenoscopy; SB small bowel; bx, biopsy; HTLV-1, Human T-lymphotropic virus Type I

* small bowel resection after medical treatment for strongyloidiasis showed poorly differentiate small bowel lymphoma

† patient underwent to a gastrojejunostomy; diagnosis was made after surgery by EGD + gastric aspirate

‡ patient presented new episode of duodenal obstruction 6 years after the initial treatment/recurrent strongyloidiasis

# initially treated with albendazole without success.

Paralytic ileus is also a potential complication of *S. stercolaris *hyperinfection [7,11,20-23]. In a recent review, Yoshida et al. have reported 25 cases of *Strongyloides*-related ileus [11]. Most of the patients were males (60%) and middle-aged, findings similar to patients with duodenal obstruction (Table 1). Despite unavailable data in the literature, it seems that obstructive gastrointestinal symptoms are more common in this specific group of patients, since the infection has no predilection for either sex or age.

Strongyloidiasis is usually associated with anemia, hypocholesterolemia and hypoalbuminemia. Eosinophilia is an inconsistent finding, present in up to 35% during the acute phase, and less frequent in patients with chronic or disseminated disease. Most patients with duodenal obstruction presented low eosinophil count indicating a chronic infection. Eosinopenia and low IgE level have been associated with a poor prognosis, in patients with disseminated disease [3,11].

Duodenal obstruction may be caused by different diseases, including tuberculosis, primary intestinal lymphoma, Crohn's disease, eosinophilic gastroenteritis and gastrointestinal stromal tumor. Despite extensive preoperative work-up, three out of the nine cases presented in Table 1, the diagnosis was made after exploratory laparotomy. Therefore, a high index of suspicion is essential for correct diagnosis of *Strongyloides*-related duodenal obstruction. The diagnosis of strongyloidiasis may be confirmed by the presence of the larvae in the stools. This is an easy performed, broadly available and inexpensive method for detection of the parasite. However, stool examination is relatively insensitive, and diagnostic yield of a single specimen is approximately 30%. The sensitivity of fecal smear could be increased to up to 60%, if five or more stool samples are examined [24]. Of note, *S. stercoralis *is the only helminth that secretes larvae in the stools. Thus, the presence of eggs in the fecal smear is unlikely.

Other methods such as duodenal aspirate or biopsy are more invasive therefore less desirable. Nevertheless, it has been shown that the examination of a duodenal aspirate for ova and larvae is the most sensitive diagnostic procedure, with a false-negative frequency of less than 10% [24,25]. Endoscopic findings include duodenal mucosal edema, erythema, hemorrhagic spots, ulcerations, and in some cases megaduodenum. Duodenal white villi is also a common endoscopic feature, and should alert the physician for the diagnosis of strongyloidiasis [25,26]. Recently, Kishimoto et al. showed that the *S. stercoralis *larvae identification in duodenal biopsies is feasible in 71% of cases [27]. In eight out of the nine cases presented in Table 1, the diagnosis was made by duodenal aspirate/biopsy, or analysis of surgical specimen. These findings confirmed the poor reliability of stool analysis for the parasite identification

In cases of disseminated infection, the parasite can be also identified in sputum, broncho-alveolar lavage, cerebrospinal fluid, skin, urine, and ascites [7]. Serology tests are indicated when the infection is suspected and the *S. stercoralis *cannot be demonstrated by the standard diagnostic evaluation. Although, indirect hemmagglutination (IHA) and indirect fluorescent antibody (IFA) test have been used, enzyme-linked immunosorbent assay (ELISA) is currently recommended because of its greater sensitivity [8,28,29]. Despite its high specificity and sensitivity, immunodiagnostic tests have certain limitations, including: (1) variable reliability in different commercial kits available, (2) falsely negative results in immunocompromised hosts, (3) the presence of anti-strongyloides antibody for a long period of time, even after successful treatment, and (4) falsely positive results due to cross-reactions with other parasitic infections such as filariasis and acute schistosomiasis [3,8].

Imaging studies are nonspecific. However, radiological abnormalities restricted to the duodenum and proximal jejunum, on CT scans and upper gastrointestinal series, should alert the surgeon to the possibility of strongyloidiasis. A unique radiographic feature of strongyloidiasis is the reflux of oral contrast into the biliary tree, possibly due to an incompetent sphincter of Oddi caused by severe inflammation of the duodenal wall [30].

Medical treatment should be achieved even in the absence of symptoms, in order to avoid the dissemination of the parasite and minimize the risk of development hyperinfection syndrome. The drug of choice for treatment of strongyloidiasis is ivermectin given at a dose of 200 mcg/kg of body weight daily for at least 2 days [3,8,31]. In cases of disseminated disease it may be necessary to prolong or repeat therapy. Albendazole and thiabendazole, are equivalent to ivermectin in efficacy. However, thiabendazole is associated with frequent and severe side effects, and has not been longer recommended for systemic infection in HIV-patients [7]. Due to a critical condition of our patient we decided to use a combination therapy of albendazole and ivermectin. This therapeutic strategy has been recommended for the treatment of disseminated strongyloidiasis with good results [3,8,25].

In patients who are not able to tolerate oral treatment, rectal administration of ivermectin or thiabendazole has been suggested [32,33]. However, recent reports have shown that serum ivermectin concentration is very low after rectal administration in patients sustaining paralytic ileus or intestinal obstruction [34,35]. No parenteral preparation of these anthelmintics is available for use in humans, although subcutaneous veterinary ivermectin has been utilized successfully in the treatment of strongyloidiasis unresponsive to standard oral therapy or when enteral administration is not feasible [34-36]. Thus, further studies assessing safety, efficacy and pharmacokinetics of parenteral ivermectin are needed in order improve the treatment and outcome of patients sustaining this unusual complication of *Strongyloides stercoralis *hyperinfection.

## Conclusion

In summary, duodenal obstruction is a rare, but potential fatal, complication of *S. stercoralis *infection. The large spectrum of clinical manifestation and lack of classic clinical syndrome make the final diagnosis of strongyloidiasis extremely difficult. Therefore a high index of suspicion, mainly in patients from endemic areas, is needed for correct and early diagnosis of this uncommon complication of *Strogyloides stercoralis *infection.

## Competing interests

The authors declare that they have no competing interests.

## Authors' contributions

All the authors participated in the admission and the care of this patient, the conception, manuscript preparation and literature search. In addition, all authors read and approved the final manuscript.

## Consent

Written informed consent was obtained from the patient's family for publication of this case report and any accompanying images. A copy of the written consent is available for review by the Editor-in-Chief of this journal.

## References

[B1] Concha R Harrington W Rogers AI Intestinal strongyloidiasis: recognition, management, and determinants of outcomes J Clin Gastroenterol 2005 39 203 211 10.1097/01.mcg.0000152779.68900.33 15718861

[B2] Mahmoud AA Strongyloidiasis Clin Infect Dis 1996 23 5 949 952 8922784 10.1093/clinids/23.5.949

[B3] Segarra-Newnham M Manifestations, diagnosis, and treatment of Strongyloides stercoralis infection Ann Pharmacother 2007 41 12 1992 2001 10.1345/aph.1K302 17940124

[B4] Olsen A van Lieshout L Marti H Polderman T Polman K Steinmann P Stothard R Thybo S Verweij JJ Magnussen P Strongyloidiasis: the most neglected of the neglected tropical diseases? Trans R Soc Trop Med Hyg 2009 103 10 967 972 10.1016/j.trstmh.2009.02.013 19328508

[B5] Genta RM Global prevalence of strongyloidiasis: critical review with epidemiologic insights into the prevention of disseminated disease Rev Infect Dis 1989 11 5 755 767 2682948 10.1093/clinids/11.5.755

[B6] Chu E Whitlock WL Dietrich RA Pulmonary hyperinfection syndrome with Strongyloides stercoralis Chest 1990 97 6 1475 1477 10.1378/chest.97.6.1475 2347234

[B7] Ramdial PK Hlatshwayo NH Singh B Strongyloides stercoralis mesenteric lymphadenopathy: clue to the etiopathogenesis of intestinal pseudo-obstruction in HIV-infected patients Ann Diagn Pathol 2006 10 4 209 214 10.1016/j.anndiagpath.2005.11.008 16844562

[B8] CDC Parisitology Diagnostic website http://www.dpd.cdc.gov/dpdx/HTML/Strongyloidiasis.htm

[B9] Harish K Sunilkumar R Varghese T Feroze M Strongyloidiasis presenting as duodenal obstruction Trop Gastroenterol 2005 26 4 201 202 16737052

[B10] Leighton PM MacSween HM Strongyloides stercoralis. The cause of an urticarial-like eruption of 65 years' duration Arch Intern Med 1990 150 8 1747 1748 10.1001/archinte.150.8.1747 2383168

[B11] Yoshida H Endo H Tanaka S Ishikawa A Kondo H Nakamura T Recurrent paralytic ileus associated with strongyloidiasis in a patient with systemic lupus erythematosus Mod Rheumatol 2006 16 1 44 47 10.1007/s10165-005-0447-1 16622724

[B12] Galimberti R Pontón A Zaputovich FA Velasquez L Galimberti G Torre A Kowalczuk A Disseminated strongyloidiasis in immunocompromised patients--report of three cases Int J Dermatol 2009 48 9 975 978 10.1111/j.1365-4632.2009.04082.x 19702983

[B13] Cohen J Spry CJ Strongyloides stercoralis infection and small intestinal lymphoma Parasite Immunol 1979 1 2 167 178 317839 10.1111/j.1365-3024.1979.tb00704.x

[B14] Zyngier FR Lemme A Pereira EG Leal BB Liberal MH Surgical perpetuation of serious infection with Strongyloides stercoralis Trans R Soc Trop Med Hyg 1983 77 3 425 10.1016/0035-9203(83)90182-7 6414126

[B15] Lee MG Terry SI Arteriomesenteric duodenal occlusion associated with strongyloidiasis J Trop Med Hyg 1989 92 1 41 45 2918578

[B16] Friedenberg F Wongpraparut N Fischer RA Gubernick J Zaeri N Eiger G Ozden Z Duodenal obstruction caused by Strongyloides stercoralis enteritis in an HTLV-1-infected host Dig Dis Sci 1999 44 6 1184 1188 10.1023/A:1026636509713 10389694

[B17] Suvarna D Mehta R Sadasivan S Raj VV Balakrishnan V Infiltrating Strongyloides stercoralis presenting as duodenal obstruction Indian J Gastroenterol 2005 24 4 173 174 16204912

[B18] Juchems MS Niess JH Leder G Barth TF Adler G Brambs HJ Wagner M Strongyloides stercoralis: a rare cause of obstructive duodenal stenosis Digestion 2008 77 3-4 141 144 10.1159/000128597 18446028

[B19] Stemmermann GN Strongyloidiasis in migrants. Pathological and clinical considerations Gastroenterology 1967 53 1 59 70 6028795

[B20] Al Maslamani MA Al Soub HA Al Khal AL Al Bozom IA Abu Khattab MJ Chacko KC Strongyloides stercoralis hyperinfection after corticosteroid therapy: a report of two cases Ann Saudi Med 2009 29 5 397 401 10.4103/0256-4947.55172 19700900 PMC2860402

[B21] Bannon JP Fater M Solit R Intestinal ileus secondary to Strongyloides stercoralis infection: case report and review of the literature Am Surg 1995 61 4 377 380 7893110

[B22] Al-Bahrani ZR Al-Saleem T Al-Gailani MA Sub-acute intestinal obstruction by Strongyloides stercolaris J Infect 1995 30 1 47 50 10.1016/S0163-4453(95)92847-2 7751666

[B23] Nonaka D Takaki K Tanaka M Umeno M Takeda T Yoshida M Haraguch Y Okada K Sawae Y Paralytic ileus due to strongyloidiasis: case report and review of the literature Am J Trop Med Hyg 1998 59 4 535 538 9790425 10.4269/ajtmh.1998.59.535

[B24] James CA Abadie SH Studies in human strongyloides II. A comparison of the efficiency of diagnosis by examination of feces and duodenal fluid Am J Clin Pathol 1954 24 1154 1158 13207044 10.1093/ajcp/24.10.1154

[B25] Lim S Katz K Krajden S Fuksa M Keystone JS Kain KC Complicated and fatal Strongyloides infection in Canadians: risk factors, diagnosis and management CMAJ 2004 171 479 484 15337730 10.1503/cmaj.1031698 PMC514646

[B26] Thompson BF Fry LC Wells CD Olmos M Lee DH Lazenby AJ Mönkemüller KE The spectrum of GI strongyloidiasis: an endoscopic-pathologic study Gastrointest Endosc 2004 59 7 906 910 10.1016/S0016-5107(04)00337-2 15173813

[B27] Kishimoto K Hokama A Hirata T Ihama Y Nakamoto M Kinjo N Kinjo F Fujita J Endoscopic and histopathological study on the duodenum of Strongyloides stercoralis hyperinfection World J Gastroenterol 2008 14 11 1768 1773 10.3748/wjg.14.1768 18350608 PMC2695917

[B28] Genta RM Predictive value of an enzyme-linked immunosorbent assay (ELISA) for the serodiagnosis of strongyloidiasis Am J Clin Pathol 1988 89 3 391 394 3348175 10.1093/ajcp/89.3.391

[B29] Lindo JF Conway DJ Atkins NS Bianco AE Robinson RD Bundy DA Prospective evaluation of enzyme-linked immunosorbent assay and immunoblot methods for the diagnosis of endemic Strongyloides stercoralis infection Am J Trop Med Hyg 1994 51 2 175 179 8074251 10.4269/ajtmh.1994.51.175

[B30] Louisy CL Barton CJ The radiological diagnosis of Strongyloides stercoralis enteritis Radiology 1971 98 3 535 541 5101578 10.1148/98.3.535

[B31] Datry A Hilmarsdottir I Mayorga-Sagastume R Lyagoubi M Gaxotte P Biligui S Chodakewitz J Neu D Danis M Gentilini M Treatment of Strongyloides stercoralis infection with ivermectin compared with albendazole: results of an open study of 60 cases Trans R Soc Trop Med Hyg 1994 88 3 344 345 10.1016/0035-9203(94)90110-4 7974685

[B32] Boken DJ Leoni PA Preheim LC Treatment of Strongyloides stercoralis hyperinfection syndrome with thiabendazole administered per rectum Clin Infect Dis 1993 16 1 123 126 8448287 10.1093/clinids/16.1.123

[B33] Tarr PE Miele PS Peregoy KS Smith MA Neva FA Lucey DR Case report: Rectal adminstration of ivermectin to a patient with Strongyloides hyperinfection syndrome Am J Trop Med Hyg 2003 68 4 453 455 12875295

[B34] Grein JD Mathisen GE Donovan S Fleckenstein L Serum ivermectin levels after enteral and subcutaneous administration for Strongyloides hyperinfection: a case report Scand J Infect Dis 2010 42 234 236 10.3109/00365540903443165 20085425

[B35] Chiodini PL Reid AJ Wiselka MJ Firmin R Foweraker J Parenteral ivermectin in Strongyloides hyperinfection Lancet 2000 355 43 44 10.1016/S0140-6736(99)02744-0 10615895

[B36] Lichtenberger P Rosa-Cunha I Morris M Nishida S Akpinar E Gaitan J Tzakis A Doblecki-Lewis S Hyperinfection strongyloidiasis in a liver transplant recipient treated with parenteral ivermectin Transpl Infect Dis 2009 11 137 142 10.1111/j.1399-3062.2008.00358.x 19144097

